# Case Report: A Novel Intronic Mutation in *AIFM1* Associated With Fatal Encephalomyopathy and Mitochondrial Disease in Infant

**DOI:** 10.3389/fped.2022.889089

**Published:** 2022-05-31

**Authors:** Qi Peng, Keze Ma, Linsheng Wang, Yinghua Zhu, Yaozhong Zhang, Chunbao Rao, Dong Luo, Zaixue Jiang, Wei Lai, Huiling Lu, Chaohui Duan, Zhongjun Zhou, Xiaomei Lu

**Affiliations:** ^1^Laboratory Department, Dongguan Children’s Hospital Affiliated to Guangdong Medical University, Dongguan, China; ^2^Department of Medical and Molecular Genetics, Dongguan Institute of Pediatrics, Dongguan, China; ^3^Key Laboratory for Children’s Genetics and Infectious Diseases of Dongguan, Dongguan, China; ^4^Pediatric Intensive Care Unit, Dongguan Children’s Hospital Affiliated to Guangdong Medical University, Dongguan, China; ^5^School of Biomedical Sciences, Li Ka Shing Faculty of Medicine, The University of Hong Kong, Pokfulam, Hong Kong SAR, China; ^6^Department of Radiology, Dongguan Children’s Hospital Affiliated to Guangdong Medical University, Dongguan, China; ^7^Laboratory of Clinical, The Sun Yat-sen Memorial Hospital, Sun Yat-sen University, Guangzhou, China

**Keywords:** *AIFM1*, whole-exome sequencing, fatal encephalomyopathy, novel intronic mutation, mitochondrial disease

## Abstract

**Background:**

The *AIFM1* gene is located on chromosome Xq26.1 and encodes a flavoprotein essential for nuclear disassembly in apoptotic cells. Mutations in this gene can cause variable clinical phenotypes, but genotype-phenotype correlations of *AIFM1*-related disorder have not yet been fully determined because of the clinical scarcity.

**Case Presentation:**

We describe a 4-month-old infant with mitochondrial encephalopathy, carrying a novel intronic variant in *AIFM1* (NM_004208.4: c.1164 + 5G > A). TA cloning of the complementary DNA (cDNA) and Sanger sequencing revealed the simultaneous presence of an aberrant transcript with exon 11 skipping (89 bp) and a normal transcript through analysis of mRNA extracted from the patient’s fibroblasts, which is consistent with direct RNA sequencing results.

**Conclusion:**

We verified the pathogenic effect of the *AIFM1* c.1164 + 5G > A splicing variant, which disturbed normal mRNA splicing. Our findings expand the mutation spectrum of *AIFM1* and point out the necessity of intronic sequence analysis and the importance for integrative functional studies in the interpretation of sequence variants.

## Introduction

The *AIFM1* (Mitochondria-Associated Apoptosis-Inducing Factor 1) gene (OMIM *300169) is located at Xq26.1 and contains 16 exons, coding for AIF (Apoptosis-Inducing Factor), a 67 kDa mitochondrial flavin adenine dinucleotide (FAD)-dependent oxidoreductase that plays a role in oxidative phosphorylation (OxPhos) and apoptosis pathway (1). This protein is present in the mitochondria and nucleus, where it participates in caspase-independent apoptosis by nuclear fragmentation (2). When apoptosis is induced, calpains and/or cathepsins cleave this protein into a soluble, proapoptogenic form that translocates to the nucleus and promotes chromosomal condensation and fragmentation (3).

*AIFM1* is known to have roles in electron transport, apoptosis, ferredoxin metabolism, reactive oxygen species generation, and immune system regulation (4). Mutations in the *AIFM1* gene have been reported to be associated with Cowchock syndrome, also known as X-linked recessive Charcot-Marie-Tooth disease-4 (CMTX-4) (5, 6), X-linked deafness-5 (DFNX5) (7), hypomyelinating leukodystrophy, and spondylometaphyseal dysplasia (H-SMD) (8). In addition, *AIFM1* mutations can cause oxidative phosphorylation defects, resulting in severe mitochondrial encephalomyopathy, which is characterized by ventriculomegaly, congenital lactic acidosis, intractable seizures, polyneuropathy, and myopathy, including hypotonia and weakness (9).

Here we describe a male infant with severe mitochondrial encephalomyopathy. We demonstrated that the patient’s state was caused by the novel hemizygous mutation c.1164 + 5G > A in the *AIFM1* gene. To our knowledge, this is the first report of the novel *AIFM1* mutation (c.1164 + 5G > A). Experiments were carried out to explore the pathogenicity of this mutation, which confirmed that the mutation could destroy the original donor splice site and lead to exon 11 skipping.

## Materials and Methods

### Clinical Presentation

The proband is a 4-month-old male infant, the second affected child of healthy non-consanguineous parents. His elder brother experienced similar symptoms and died 5 years ago at the age of 4 months. The proband was born at term with a birth weight of 2.53 kg following a normal pregnancy. On the second day after birth, the patient presented with frequent apneas and cyanosis, and was transferred to our hospital.

He presented with hypotonia and marked muscle weakness. His blood oxygen level was reduced to 77% (normal range for newborns: 93–100%), and the heart rate dropped to 110–120 beats/min (normal range for newborns:120–140 beats/min). The peripheral blood glucose fluctuated between 4.0 and 6.0 mmol/L (normal range: 3.9–6.1 mmol/L) during this period. Nasal continuous positive airway pressure (NCPAP) was used to maintain airway patency. A CT scan of the brain revealed an existing subarachnoid hemorrhage. Laboratory investigations revealed plasma lactate concentration fluctuated between normal and abnormal. Blood acylcarnitine analysis and urinary organic acid profiling showed no abnormality. Laboratory tests revealed total bilirubin (TBIL) of 170.8 μmol/L (normal range: 0–23 μmol/L) and direct bilirubin (DBIL) of 11.1 μmol/L (normal range: 0–4 μmol/L), and renal functions were basically normal.

After being hospitalized for 5 days in our hospital, the child was transferred to a provincial hospital for more than 2 months. During this period, the child underwent a number of examinations and no clear cause was determined. At said hospital, no pathogenic mutations or copy number variations were found in genes related to the disease.

At roughly 4 months old, the child was sent to the emergency department of our hospital due to spit-up and cyanosis at home. He had gone into a sudden respiratory and cardiac arrest. After rescue, the child’s complexion gradually turned rosy, and his spontaneous heartbeat was restored. Considering that the child’s breathing was unstable after successful cardiopulmonary resuscitation, advanced life support treatment was still needed, so he was transferred to PICU under the condition of tracheal intubation and resuscitation bag positive pressure ventilation for further monitoring and treatment.

Later, despite the use of non-invasive respiratory support, the child still had repeated apneas, accompanied by mental fatigue, drowsiness, and sudden hypotension. The lactic acid was always high, fluctuating at 5–12.9 mmo/L (normal range: 0.5–2.2 mmol/L), showing a progressive increase.

**FIGURE 1:**
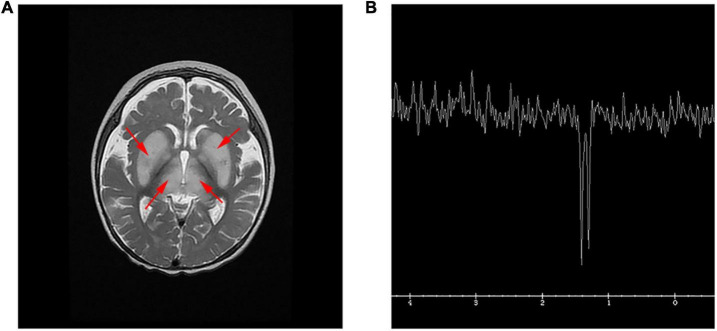
MRI and MRS in the patient. **(A)** T2 weighted image (T2WI) showed symmetrical hyperintensity in the basal ganglia and thalamus, and a small amount of subdural effusion in the forehead. **(B)** Single voxel MRS of the left basal ganglia. The lactate peak, clearly visible as an inverted doublet at TE = 144 ms.

A brain magnetic resonance imaging (MRI) examination was performed and revealed a bilateral hyperintensity of basal ganglia (Figure 1A). Hydrocephalus was suggested by a moderately enlarged ventricle system, centered on each side of the ventricle and a widened extracerebral space in the temporal part of both sides. The patient’s lactate peak was distinctly discernible as an inverted doublet at TE = 144 ms by single voxel magnetic resonance spectroscopy (MRS) of the left basal ganglia (Figure 1B). Multiple basic symmetrical abnormal signals, combined with MRS can be seen in the bilateral basal ganglia, brainstem, thalamus, frontal lobe, parietal lobe, bilateral limbic lobe, and temporal cortex, suggesting mitochondrial encephalopathy. Finally, the proband died due to respiratory failure after the parents ceased treatment.

### Whole Exome Sequencing and Bioinformatic Analyses

The peripheral blood samples were obtained from the patient and his family members after informed consent was obtained. Whole Exome Sequencing (WES) was performed to analyze the coding exons and the exon-intron boundaries of protein-coding genes by the Novogene Bioinformatics Institute (Tianjin, China). The American College of Medical Genetics and Genomics (ACMG) guidelines were also used to classify this variant. The 3D models of the normal and mutant protein are predicted by the I-TASSER server^1^ and visualization was carried out by PyMOL.

### mRNA Sequencing

RNA sequencing was conducted by the Novogene Bioinformatics Institute (Beijing, China). Preparation of sample is followed by the RNA library preparation. RNA library is formed by polyA capture (or rRNA removal) and reverse transcription of cDNA. Illumina PE150 technology is employed to sequence the sample and the final stage involves the bioinformatics analysis.

### Muscle and Skin Biopsy

Quadricep muscle and skin biopsies were obtained from the patient. Age- and gender-matched specimens of normal human muscle and skin were obtained from a patient during surgery. For the use of biomedical studies, fibroblasts were cultured and harvested from the biopsies.

### Reverse Transcription-Polymerase Chain Reaction and TA Clone Sequencing

We preformed reverse transcription-polymerase chain reaction (RT-PCR) on mRNA extracted from the patient’s fibroblasts in order to evaluate the potential effect of this variant on splicing. The cDNA obtained was amplified by PCR, using a set of primers: the forward primer aligning on exon 10 (5′-CAGCAACTGGACCATGGAAA-3′) and the reverse primer aligning on exon 12 (5′-GCTCTGCATTTACCCGGAAG-3′) of *AIFM1* gene. The PCR products were gel-purified and ligated into the pMD19-T vector and transformed into *E. coli* DH5α. The colonies were picked and screened by PCR, and the positive clones were sent for sequencing (Sangon Biotech, Shanghai, China).

### Real-Time Quantitative Polymerase Chain Reaction

The relative mRNA expression levels of *AIFM1* in the fibroblasts of the patient and control normal individual were compared by real-time quantitative polymerase chain reaction (RT-qPCR). Four pairs of primers were designed to detect the mRNA level of *AIFM1*. Each experiment was performed in triplicate, and expression data were normalized to β-actin as an internal reference gene. The 2^–ΔΔCt^ method was used for quantification by comparing the Ct values.

### Western Blot Analysis

Fibroblasts were trypsinied, centrifuged at 1000 *g* for the duration of 5 min, followed by solubilizing in RIPA buffer with protease inhibitors. For muscle tissues, ice-cold lysis buffer was rapidly added to the samples, followed by homogenizing with an electric homogenizer and rinsing the blade twice with another 2 × 300 μL lysis buffer. The samples were then kept at 4°C for 2 h with continual agitation. Immunoblot analysis was performed with the ECL-chemiluminescence kit in accordance with manufacturer’s protocol. A mouse monoclonal anti-GAPDH (4A Biotech Co., Ltd., Shanghai, China, Cat. No. 4ab030004) and mouse monoclonal anti-AIFM1 antibody binding to the C-terminus of human AIF (Boster Biological Technology, Wuhan, China, Cat. No. M01571-1) were used. The HRP-conjugated Affinipure Goat Anti- Rabbit IgG (H + L) was used as secondary antibody (Proteintech, Illinois, IL, United States, Cat. No. SA00001-2).

### Immunofluorescence

For immunofluorescence, cells were fixed in 4% paraformaldehyde and permeabilized in 0.25% Triton X-100. Subsequently, cells were blocked and immunostained with antibodies in 5% goat serum and treated with 4′,6-Diamidino-2-phenylindole (DAPI) staining solution (Beyotime Biotechnology, Shanghai, China) before mounting. Anti-TOMM20 (Millipore, Bedford, MA, United States) was used at 1/200 dilution for mitochondria localization. Images were visualized using Nikon Eclipse Ti-S Phase Contrast Fluorescence Inverted Microscope (Nikon, Tokyo, Japan).

### NAD^+^ and Cytochrome C Oxidase Measurement

The level of NAD^+^ in fibroblasts was determined using NAD^+^ /NADH assay kit (Beyotime, S0175, Shanghai, China) with WST-8 according to the manufacturer’s instructions. Human Cytochrome C Oxidase (COX) of cell culture supernatant were measured using the Human COX ELISA kit (RX105095H, Ruixin Biotech, Quanzhou, Fujian, China) according to instruction of the manufacturer.

## Results

### Whole Exome Sequencing Analysis

**FIGURE 2:**
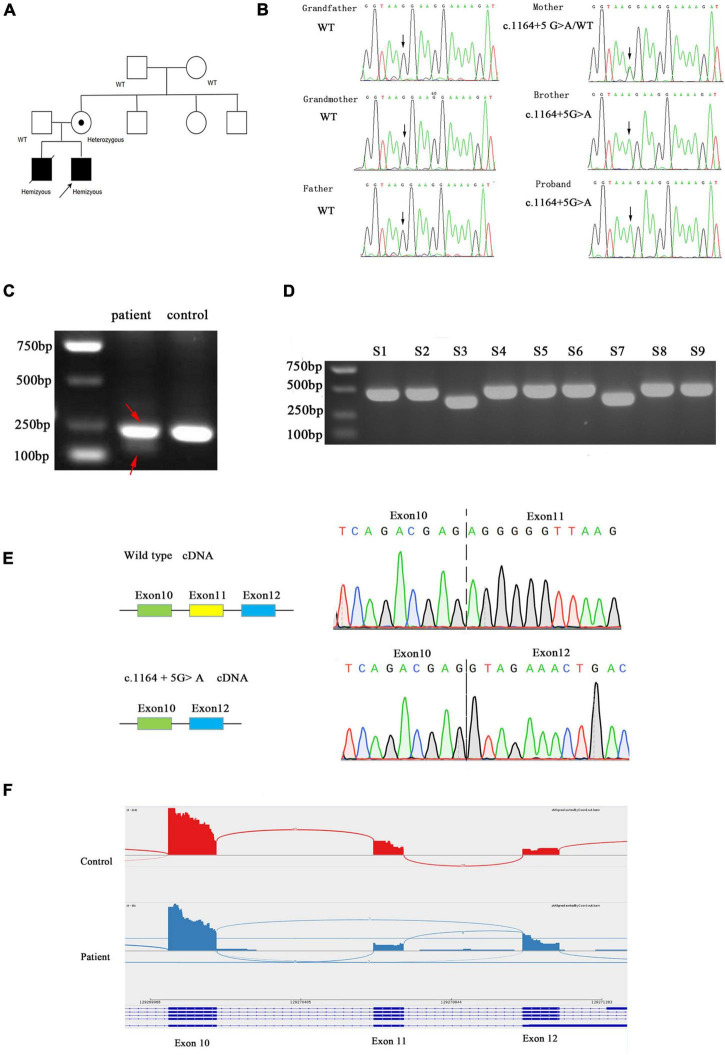
**(A)** Family pedigree. Black symbols represent affected persons and symbols with a dot, carriers. The proband is indicated by an arrow. **(B)** Chromatograms for the mutations confirmed by Sanger sequencing. **(C)** Agarose gel electrophoresis of the transcripts generated by cDNA amplification of the patient and control, with adjacent schematic representation of the resulting splicing events. The PCR product of patient was marked by red arrow. **(D)** Agarose gel electrophoresis of cloning PCR. S1–S9 represent the different clones picked. **(E)** Sanger sequencing electropherogram of gel-purified fragments for the upper band and lower band from the patient cDNA samples. **(F)** AIFM1 Sashimi plot of RNA extracted from patient’s and control’s fibroblasts.

In the proband, WES identified a maternally inherited hemizygous variant in intron 11 of the *AIFM1* gene, c.1164 + 5G > A, on the X chromosome. Sanger sequencing confirmed that this mutation was also present in his elder brother who died 5 years ago due to similar symptoms. His mother is a heterozygous carrier of the mutation, but maternal grandparents don’t have this mutation. Therefore, this is a *de novo* mutation that occurred in the mother and passed down to the two sons (Figures 2A,B).

c.1164 + 5G > A is absent from dbSNP version 147, 1000 genomes project, ExAC, HGMD, and Clinvar databases and not found in the literature. Computational predictions using SpliceAI, HSF, and MatEntScan did not predict intronic c.1164 + 5G > A to have an impact on splicing.

### Functional Analysis

We evaluated the functional significance of the c.1164 + 5G > A mutation because it was missing from databases and co-segregated with disease phenotype in the family. The control fibroblasts produced a single 236 bp product by reverse-transcription PCR targeting exon 10 and exon12 of the *AIFM1* gene, whereas the patient’s fibroblasts produced two bands (236 and 147 bp) (Figure 2C). In order to clarify the two bands, the PCR products were TA cloned and screened by cPCR, then two sizes of products were clearly seen (Figure 2D). Sanger sequencing showed that the shorter product on the patient’s fibroblasts lacked exon 11 (89 bp) (Figure 2E). The novel splice variant in *AIFM1* led to exon 11 skipping. The effect of this intron mutation on splicing was confirmed through RNA-sequencing (Figure 2F).

**FIGURE 3:**
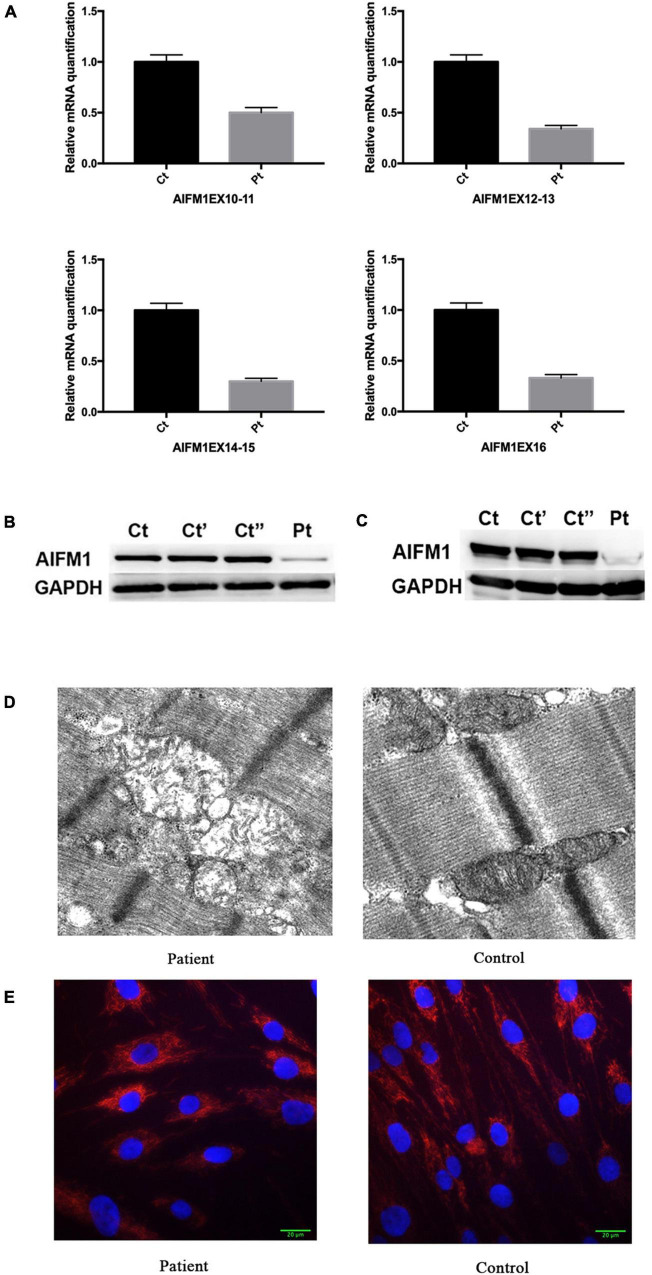
**(A)** Relative quantification of mRNA levels for AIFM1 in controls and patient’s fibroblasts using different primers. The results of mRNA are the average of the values assessed after three reaction tests. **(B)** Western blot analysis in controls and patient’s fibroblasts using AIF antibody. GAPDH was used as loading control. The patient’s sample shows a clear reduction in the AIF amount compared with the control lines. **(C)** Western blot analysis in controls and patient’s muscles using AIF antibody. GAPDH was used as loading control. **(D)** Electron microscopy of quadriceps muscle demonstrating large, irregularly shaped mitochondria, including one with concentric cisternae. **(E)** TOMM20 staining of the patient’s and control fibroblast. DAPI was used to mark the nucleus.

The RT-qPCR analysis using four pairs of primers targeting the sequence after the mutation site showed that the *AIFM1* mRNA levels were significantly decreased in the patient’s fibroblasts (Figure 3A). The detrimental effect of the identified variant was also confirmed using western blot analysis, which showed a highly reduced amount of AIF protein in the patient’s fibroblasts (Figure 3B) and muscle (Figure 3C) compared with controls. These findings highlighted the novel variant’s potential pathogenic effect, as the intronic mutation leads to reduced AIF1 expression, likely due to incomplete penetrance of the splicing defect, allowing minimal production of wildtype transcript.

Electron microscopy analysis of a muscle biopsy from the patient revealed large mitochondria with insufficient fusion of the inner mitochondrial membrane. The mitochondria were contorted and irregularly shaped, with no inclusions (Figure 3D). Mitochondrial fragmentation was also noted in the patient’s fibroblast by staining the mitochondrial protein TOMM20 (Figure 3E).

**FIGURE 4:**
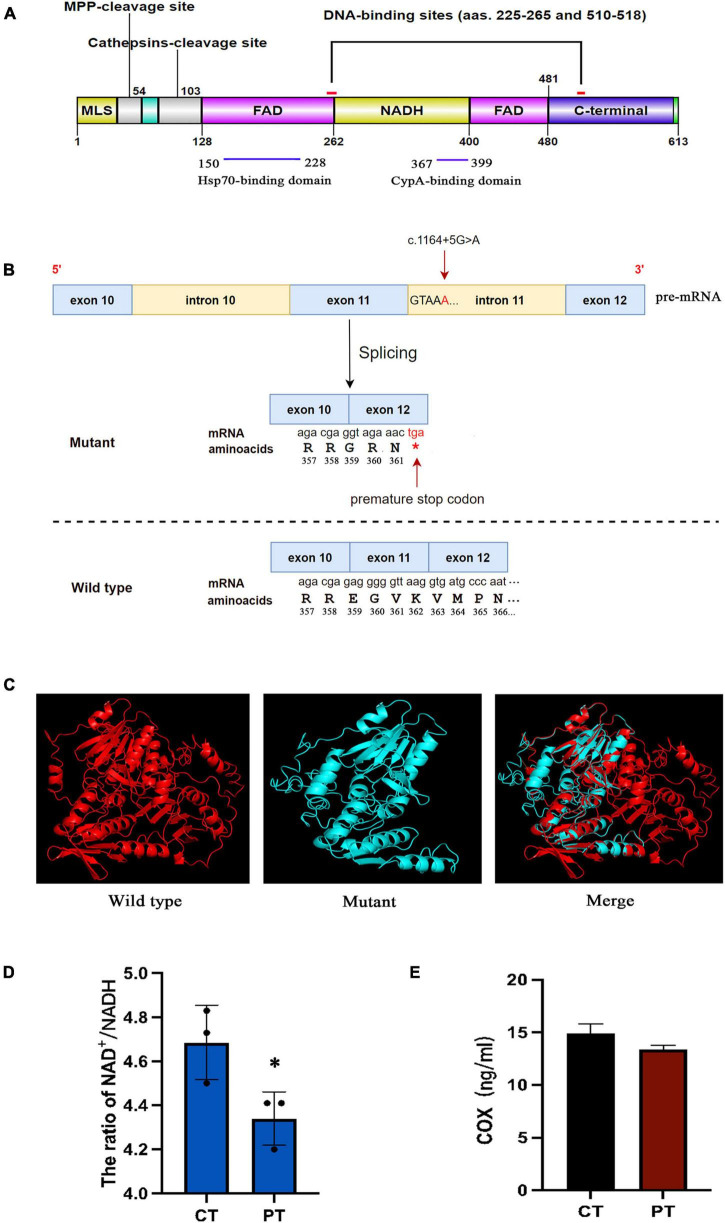
**(A)** Schematic model representing the AIF protein. AIF is a flavoprotein (with an oxidoreductase enzymatic activity) containing a FAD-bipartite domain (amino-acids 128–262 and 401–480), a NADH-binding motif (amino-acids 263–400), and a C-terminal domain (amino-acids 481–608) where the proapoptotic activity of the protein resides. In addition, it has a Mitochondria Localization Sequence (MLS, amino-acids 1–41) placed in its N-terminal region. AIF also possesses two DNA-binding sites, which are located in amino-acids 255–265 and 510–518, respectively. **(B)** The mutant causes a frameshift resulted in the change of amino acid coding after R358, and the early termination codon was generated at the position of amino acid 362. **(C)** Protein structure modeling of wild-type and mutated AIFM1. A part of the amino acid sequence has been eliminated in the mutated protein compared to the wild type protein. **(D)** The NAD^+^/NADH ratios in fibroblasts of patient and control. **(E)** The human COX of cell culture supernatant in patient and control. *Statistically significant difference at *p* < 0.05.

The AIF protein contains three major domains: a FAD-binding domain (residues 128–262 and 401–480), a NADH-binding domain (residues 263–400), and a C-terminal domain (residues 481–608) (Figure 4A). The non-coding mutation identified in the present study was located at 5 bp of intron 11 which was close to exon 11 (Figure 4B). It was located at the 5′ splice site and was verified to cause exon 11 skipping, which competes with the wild-type splicing donor site, resulting in two coexisting transcript populations, the mutant, and the wild-type form. The mutant causes a frameshift resulted in the change of amino acid coding after R358, and the early termination codon was generated at the position of amino acid 362 (the full-length of AIF is 613 amino acids) (Figure 4B). Computational modeling for wild-type and mutant AIF were performed using I-TASSER to execute the 3D structure. As shown in Figure 4C, part of the NADH domain, the second FAD region and C-terminal are deleted in the mutant *AIFM1*, which might change its protein stability. The NAD^+^/NADH ratio is decreased in patient’s fibroblasts (Figure 4D), while the COX of cell culture supernatant showed no difference between the patient and control (Figure 4E).

## Discussion

This study describes a patient with fatal encephalomyopathy and mitochondrial disease that carried a novel intronic variant in the *AIFM1* gene (NM_004208.4: c.1164 + 5G > A). *AIFM1* gene has already been linked to mitochondrial encephalomyopathy, while intronic mutations of *AIFM1* have not been reported yet (4). To the best of our knowledge, this study is the first to report the variant, which is not included in the HGMD, 1000 Genomes Project and dbSNP147 databases.

The discovery of an intronic mutation in the AIFM1 gene prompted us to evaluate AIFM1 mRNA splicing in patient fibroblasts using RNASeq. We provided the first experimental characterization of this novel variant and elucidated its impact on RNA splicing. There are two possible outcomes for mRNAs carrying premature termination codons: non-sense-mediated mRNA decay (NMD) (10, 11) or translation to truncated proteins (12). NMD is an evolutionarily conserved quality assurance pathway found in eukaryotic cells. It has the function of examining mRNA for any potential errors, eliminating any error-containing transcripts, and regulating the amount of non-mutated transcript in the transcriptome (13). According to the mammalian non-sense-mediated mRNA decay (NMD) rule that stop codons located > 50–55 nucleotides upstream of the 3′ most splice-generated exon-exon junction usually trigger NMD in mammals (14), this mutation probably results in degradation by non-sense-mediated decay.

Alternative splicing is one of the important mechanisms in regulating gene expression, splice sites (5′ and 3′), the branch site and the polypyrimidine sequence are the key splicing signals that have major roles in the splicing of pre-mRNA (15). Splicing mutations have been identified in a number of different genes and shown to have a variety of consequences, including exon skipping and intron retention (16). Most of these splicing mutations occur within or close to conserved consensus donor (+1/+2) or acceptor (−1/−2) splice sites (17). However, mutations remote from the splice donor or acceptor sites have also been shown to lead to aberrant splicing in a number of reports (18–20). Thus, intronic mutations particularly within the splice sites (∼20 bp) must be considered more readily when evaluating sequence variants, especially when tributary mutation in coding regions or canonical splice sites cannot be found. This mutation was not recognized until this case study despite the subject’s brother having the same mutation and dying of the same disease 5 years previous. Unfortunately, due to the lack of regular intronic sequencing, and atypical mitochondrial encephalopathy presentation in the early stage, with mild changes in lactic acid meant the subjects were not diagnosed until after death.

Pathogenic intronic mutations can be detected by WGS and analyzed by RNA-Seq. This combined strategy makes it possible to unravel cases with more arduous molecular causes and will indisputably improve the mutation identification methods in the future. Due to the limitations of bioinformatic tools such as the splicing predictor, our study suggested that functional analysis is required to determine the pathogenicity of mutations. As a result, our research is the first functional validation report of a novel splicing mutation in *AIFM1* associated with fatal encephalomyopathy and mitochondrial disease.

In conclusion, our findings show that a unique intronic mutation of the *AIFM1* gene affects splicing, expanding the mutation spectrum of *AIFM1* and emphasizing the importance of intronic sequence analysis and integrative functional research in the elucidation of sequence variants.

## Data Availability Statement

The datasets presented in this study can be found in online repositories. The names of the repository/repositories and accession number(s) can be found below: NCBI (accession: NM_004208.4).

## Ethics Statement

This study was authorized by and carried out in compliance with the protocol of the Institutional Medical and Ethics Committee of Dongguan Children’s Hospital Affiliated to Guangdong Medical University. Written informed consent to participate in this study was provided by the participants’ legal guardian/next of kin.

## Author Contributions

XL and ZZ designed the study. KM, LW, YHZ, YZZ, CR, DL, ZJ, and HL carried out experimental work. WL performed the MRI studies. QP and XL prepared the first draft of the manuscript. CD revised the manuscript. All authors approved the final manuscript.

## Conflict of Interest

The authors declare that the research was conducted in the absence of any commercial or financial relationships that could be construed as a potential conflict of interest.

## Publisher’s Note

All claims expressed in this article are solely those of the authors and do not necessarily represent those of their affiliated organizations, or those of the publisher, the editors and the reviewers. Any product that may be evaluated in this article, or claim that may be made by its manufacturer, is not guaranteed or endorsed by the publisher.

## Footnotes

1
https://zhanglab.dcmb.med.umich.edu/I-TASSER/
